# *Rothia aeria* pneumonia in an immunocompetent patient: A novel case study

**DOI:** 10.1002/rcr2.843

**Published:** 2021-09-21

**Authors:** Kei Sonehara, Taisuke Araki, Masayuki Hanaoka

**Affiliations:** ^1^ First Department of Internal Medicine Shinshu University School of Medicine Matsumoto City Japan

**Keywords:** agranulocytosis, case report, pneumonia, *R. aeria*, trimethoprim/sulfamethoxazole

## Abstract

An 80‐year‐old woman with no lung disease or autoimmune disease presented with a productive cough, lasting for 2 months. Chest computed tomography revealed a transbronchial dispersal shadow in the left upper lobe, and sputum culture showed Gram‐positive rods. The identified causative organism was *Rothia aeria*, and thus, she was treated with oral trimethoprim/sulfamethoxazole (TMP–SMX). Eleven days after initiating TMP–SMX treatment, she returned with a complaint of dyspnoea. While the sputum culture revealed normal flora, the patient's condition was diagnosed as bronchitis during *R. aeria* pneumonia treatment; therefore, she was hospitalized. Five days after admission, her laboratory findings revealed agranulocytosis, caused by an adverse event of TMP–SMX. Her neutrophil count increased after discontinuing TMP–SMX treatment. Bronchitis was alleviated with intravenous antibiotic administration, and she was discharged on Day 8. We report a rare case of *R. aeria* pneumonia in an immunocompetent patient.

## Graphical abstract

*Rothia* species are indigenous bacteria in the oral cavity and upper gastrointestinal tract. They rarely cause serious infections. Here, we report a rare case of pneumonia caused by *Rothia aeria* in an immunocompetent patient.

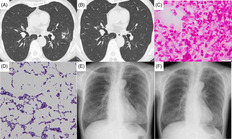

## INTRODUCTION

Pathogenic aerobic actinomycetes include mycolic acid‐containing actinomycetes such as *Nocardia*, *Rhodococcus*, *Gordonia* and *Tsukamurella* and mycolic acid‐free actinomycetes such as *Rothia and Nocardiopsis*. Actinomycetes are involved in diabetes and chronic respiratory diseases. They are also reported to affect healthy subjects. *Rothia* species are indigenous bacteria in the oral cavity and upper gastrointestinal tract. They rarely cause serious infections such as gut translocation, mucositis and catheter‐related infection, even in an immunocompromised host.^1^ We report a rare case of pneumonia caused by *Rothia aeria* in an immunocompetent patient.

## CASE REPORT

**FIGURE 1:**
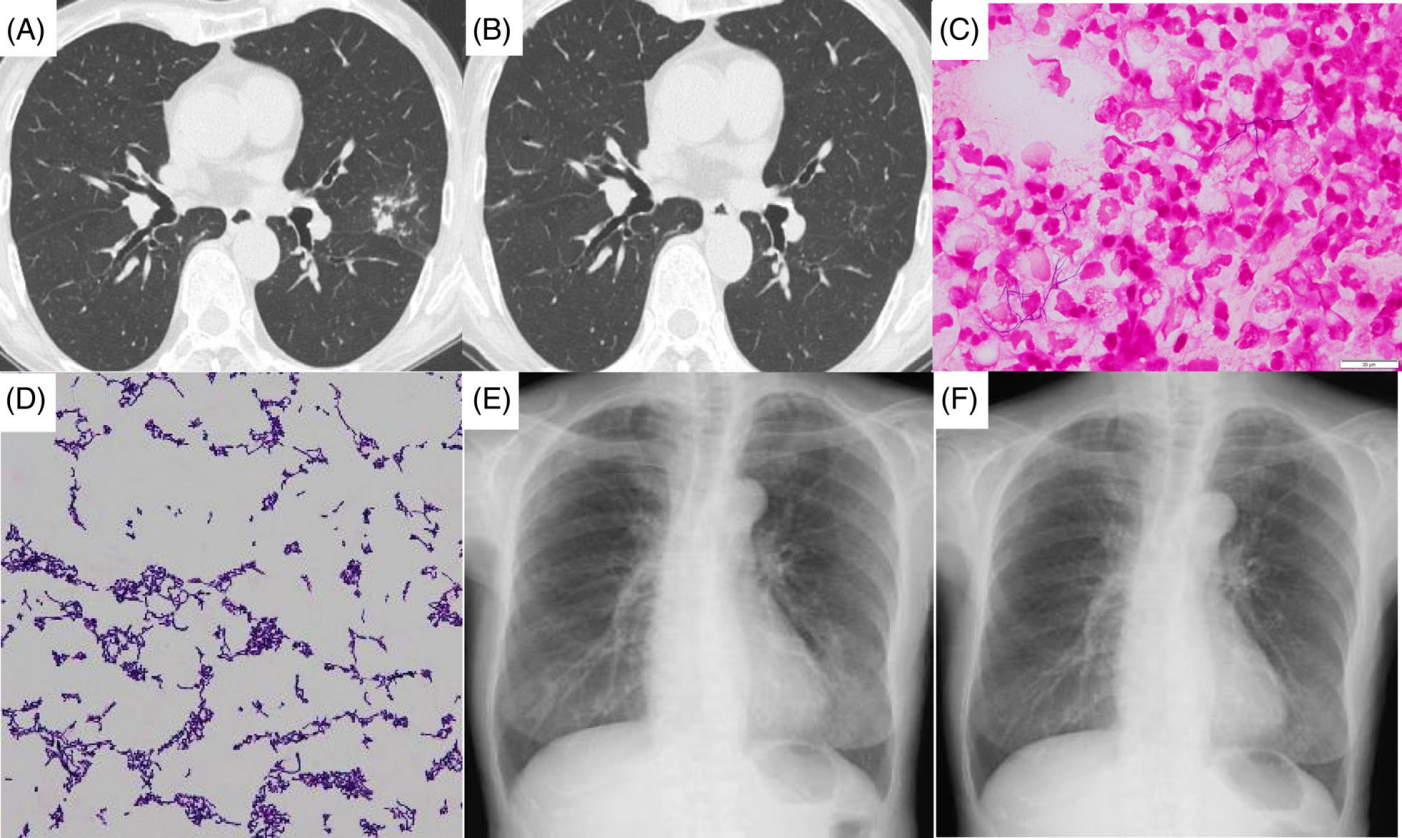
(A) Chest computed tomography (CT) before the diagnosis of *Rothia aeria* showing a transbronchial dispersal shadow in the left upper lobe of the lung. (B) Chest CT performed on Day 61 after the onset of bronchitis did not reveal any abnormalities. (C) Gram stain of the sputum sample showing Gram‐positive rods. (D) Gram stain of the sputum sample under high‐power magnification showing many Gram‐positive rods that were later identified as *R. aeria*. (E) Chest radiography on the day of treatment commencement showed infiltrative opacities in the lower fields of the left lung. (F) Chest radiography on Day 61 after the onset of bronchitis did not show any abnormalities

An 80‐year‐old woman presented to the hospital with a productive cough lasting for 2 months. She was a never‐smoker and had a history of dyslipidaemia, hyperuricaemia and pancreatic cysts. On physical examination, she was afebrile, had an arterial blood oxygen saturation of 98% (on room air) and no rales. Her oral health was good. Chest computed tomography (CT) of the lung revealed a transbronchial dispersal shadow in the left upper lobe (Figure 1A). Laboratory findings are shown in Table 1. No abnormal findings were noted. After collecting her sputum sample, the patient was treated with an expectorant. One month later, while her symptoms did not exacerbate, a microscopic analysis of the sputum culture revealed the presence of Gram‐positive rods (Figure 1C,D). Chest radiography showed infiltrative opacities in the lower fields of the left lung (Figure 1E). She received oral trimethoprim/sulfamethoxazole (TMP–SMX) because *Nocardia* and *Actinomyces* species were suspected based on the bacterial morphotype. TMP–SMX was administered at a daily dose of 320 mg for TMP and 1600 mg for SMX. Mass spectrometry revealed *R. aeria* as the causative agent. *R. aeria* was cultivated during two sputum culture tests, the samples for which were collected on different days before diagnosis. *R. aeria* is susceptible to benzylpenicillin, ampicillin, cefazolin, ceftriaxone, meropenem, erythromycin, clindamycin, minocycline, levofloxacin and TMP–SMX. Eleven days after initiating TMP–SMX treatment, she returned to the hospital with a chief complaint of dyspnoea. Physical examination revealed a Glasgow Coma Scale score of E4V5M6, a body temperature of 36.9°C, blood pressure of 116/72 mm Hg, regular pulse of 96 beats/min, respiratory rate of 20 breaths/min and arterial blood oxygen saturation of 96% (on room air). Analysis of haematological parameters revealed a white blood cell count of 5100/μl, neutrophil count of 3407/μl, haemoglobin level of 14.2 g/dl, platelet count of 17.1 × 10^4^/μl and C‐reactive protein level of 4.64 mg/dl. No bacteria were observed on Gram staining of the sputum, and the causative bacterium was unknown. Her condition was diagnosed as bronchitis during *R. aeria* pneumonia treatment, and antibiotic treatment commenced on hospitalization. On Day 5 after admission, her dyspnoea was relieved with the intravenous administration of antibiotics; however, her neutropenia (420 cells/μl) worsened. Therefore, oral TMP–SMX was discontinued. On Day 8 after admission, the neutrophil count increased to 1491/μl and she was discharged. On Day 61, she reported no respiratory symptoms. Chest radiography and chest CT did not reveal any abnormalities (Figure 1B,F).

**TABLE 1:** Laboratory findings before the treatment of *Rothia aeria* pneumonia

Haematology	Blood chemistry
White blood cells 5100/μl	Total protein 6.8 g/dl
Neutrophils 52.7%	Albumin 4.3 g/dl
Lymphocytes 28.7%	Urea nitrogen 13.7 mg/dl
Monocytes 9.1%	Creatinine 0.65 mg/dl
Eosinophils 8.3%	Creatinine kinase 92 IU/L
Basophils 1.2%	Sodium 143 mEq/L
Red blood cells 439 × 10^4^/μl	Potassium 4.3 mEq/L
Haemoglobin 13.6 g/dl	Chloride 106 mEq/L
Haematocrit 40.9%	C‐reactive protein 0.04 mg/dl
Platelets 19.0 × 10^4^/μl	

## DISCUSSION

We present a rare case of *R. aeria*‐related pneumonia in an immunocompetent patient. The *Rothia* species belongs to the family *Micrococcaceae*. There are seven species in the genus: *R. aeria*, *Rothia amarae*, *Rothia dentocariosa*, *Rothia endophytica*, *Rothia mucilaginosa*, *Rothia nasimurium and Rothia terrae*. *Rothia* species are Gram‐positive, aerobic and non‐motile and appear coccoidal, coccobacillary or filamentous. *R. aeria* was first isolated from the Russian Mir Space Station in 2004.^2^ Respiratory tract infections caused by *R. aeria* are rare, with only two reported cases. The first case was acute bronchiolitis secondary to etanercept administration for rheumatoid arthritis, while the second case was cavity pneumonia that developed secondary to the administration of azathioprine and steroids for neurosarcoidosis.^3^, ^4^ Unlike these two cases, the patient in the present case was on medications for dyslipidaemia and hyperuricaemia but did not receive immunosuppressive medications. This is a rare case to document *R. aeria* as the causative bacterium of pneumonia in an immunocompetent patient. *R. aeria* is susceptible to several antibiotics.^4^ In this case, *R. aeria* was responsive to β‐lactams, macrolides and quinolones. TMP–SMX was the selected treatment because the bacterium belonged to *Nocardia* species, identified based on the morphotype of the Gram‐positive rods in the sputum. As *R. aeria* was not cultured in the sputum during the onset of secondary bronchitis, TMP–SMX was effective against *R. aeria* pneumonia. The development of secondary bronchitis despite TMP–SMX treatment was likely caused by the increased susceptibility to infection owing to agranulocytosis, an adverse event of TMP–SMX. Although dyspnoea was exacerbated, the possibility of pneumonia refractory to TMP‐SMX and drug‐induced lung disease was excluded because no bacteria were observed on Gram staining of the sputum, and no new infiltrative opacities were observed on chest radiography. The neutrophil count at admission was within the normal range because of developing secondary bronchitis when the neutrophil count was decreasing due to agranulocytosis. A previous study reported that the incidence rate of neutropenia owing to TMP–SMX and median days to onset were 15% and 10 days, respectively.^5^ In the present case, agranulocytosis was resolved by discontinuing TMP–SMX treatment, but the administration of granulocyte colony‐stimulating factor was considered when the neutrophil count did not increase. The duration of antibiotic treatment for *R. aeria* pneumonia is uncertain. Pneumonia recurrence has not been observed, but the patient will be followed up in the future.

## CONFLICT OF INTEREST

None declared.

## AUTHOR CONTRIBUTION

Kei Sonehara drafted and reviewed the manuscript. All authors collected the data.

## ETHICS STATEMENT

Appropriate written informed consent was obtained for publication of this case report and accompanying images.
